# Seasonal Dynamics of Pelagic Mycoplanktonic Communities: Interplay of Taxon Abundance, Temporal Occurrence, and Biotic Interactions

**DOI:** 10.3389/fmicb.2020.01305

**Published:** 2020-06-26

**Authors:** Stefanos Banos, Deisy Morselli Gysi, Tim Richter-Heitmann, Frank Oliver Glöckner, Maarten Boersma, Karen H. Wiltshire, Gunnar Gerdts, Antje Wichels, Marlis Reich

**Affiliations:** ^1^Molecular Ecology Group, University of Bremen, Bremen, Germany; ^2^Department of Computer Science, Interdisciplinary Center of Bioinformatics, University of Leipzig, Leipzig, Germany; ^3^Swarm Intelligence and Complex Systems Group, Faculty of Mathematics and Computer Science, University of Leipzig, Leipzig, Germany; ^4^Center for Complex Networks Research, Northeastern University, Boston, MA, United States; ^5^Microbial Ecophysiology Group, University of Bremen, Bremen, Germany; ^6^Alfred-Wegener-Institut Helmholtz-Zentrum für Polar- und Meeresforschung, Bremerhaven, Germany; ^7^Department of Life Sciences and Chemistry, Jacobs University Bremen gGmbH, Bremen, Germany; ^8^MARUM, Center for Marine Environmental Sciences, University of Bremen, Bremen, Germany; ^9^Alfred-Wegener-Institut Helmholtz-Zentrum für Polar- und Meeresforschung, Biologische Anstalt Helgoland, Helgoland, Germany; ^10^FB2, University of Bremen, Bremen, Germany; ^11^Alfred-Wegener-Institut Helmholtz-Zentrum für Polar- und Meeresforschung, Wattenmeerstation, List, Germany

**Keywords:** phytoplankton, zooplankton, marine fungi, food web structure, microbial loop, pattern, mycoloop, zoosporic fungi

## Abstract

Marine fungi are an important component of pelagic planktonic communities. However, it is not yet clear how individual fungal taxa are integrated in marine processes of the microbial loop and food webs. Most likely, biotic interactions play a major role in shaping the fungal community structure. Thus, the aim of our work was to identify possible biotic interactions of mycoplankton with phytoplankton and zooplankton groups and among fungi, and to investigate whether there is coherence between interactions and the dynamics, abundance and temporal occurrence of individual fungal OTUs. Marine surface water was sampled weekly over the course of 1 year, in the vicinity of the island of Helgoland in the German Bight (North Sea). The mycoplankton community was analyzed using 18S rRNA gene tag-sequencing and the identified dynamics were correlated to environmental data including phytoplankton, zooplankton, and abiotic factors. Finally, co-occurrence patterns of fungal taxa were detected with network analyses based on weighted topological overlaps (wTO). Of all abundant and persistent OTUs, 77% showed no biotic relations suggesting a saprotrophic lifestyle. Of all other fungal OTUs, nearly the half (44%) had at least one significant negative relationship, especially with zooplankton and other fungi, or to a lesser extent with phytoplankton. These findings suggest that mycoplankton OTUs are embedded into marine food web chains via highly complex and manifold relationships such as parasitism, predation, grazing, or allelopathy. Furthermore, about one third of all rare OTUs were part of a dense fungal co-occurrence network probably stabilizing the fungal community against environmental changes and acting as functional guilds or being involved in fungal cross-feeding. Placed in an ecological context, strong antagonistic relationships of the mycoplankton community with other components of the plankton suggest that: (i) there is a top-down control by fungi on zooplankton and phytoplankton; (ii) fungi serve as a food source for zooplankton and thereby transfer nutrients and organic material; (iii) the dynamics of fungi harmful to other plankton groups are controlled by antagonistic fungal taxa.

## Introduction

The marine biological pump plays a central role in Earth’s ecosystems. The core of this biological pump is the carbon cycle. In this process, carbon is drawn down from the atmosphere into the ocean by phytoplankton-driven photosynthesis. The generated organic carbon is largely degraded and taken up by microorganisms and subsequently transferred to the ocean’s depths or to higher trophic levels via the food web (Turner, 2015). The current valid framework of the microbial loop includes bacteria and archaea as well as eukaryotic protists (Worden et al., 2015). Remarkably, marine fungi are still largely excluded, despite the increasing evidence that they can assimilate and decompose essential amounts of phytoplankton-derived organic matter (Gutierrez et al., 2011; Cunliffe et al., 2017), influence phytoplankton population dynamics (Gutierrez et al., 2016), or may act as trophic link between phytoplankton and zooplankton via a marine mycoloop (Amend et al., 2019) analogous to the one described for freshwater systems (Kagami et al., 2014).

Marine microbial plankton, including fungi, are exposed to a highly fluctuating environment, which is reflected in changing diversity and structure (Gilbert et al., 2012; Lucas et al., 2016; Taylor and Cunliffe, 2016; Teeling et al., 2016; Duan et al., 2018). The dynamics within marine microbial communities can be described by different patterns (Needham et al., 2013), ranging from relatively small variations around an average to rapid increases and decreases within a very short time. These dynamics can result in dramatic changes in the overall microbial abundance. Nevertheless, despite these fluctuations, the functionality of the microbial community seems to be maintained (Wittebolle et al., 2009; Werner et al., 2011). Although it has long been assumed that solely the abundant species of a community are relevant for maintaining its functionality and stability, we now know that the rare fraction is also involved (Huber et al., 2007; Hernandez-Raquet et al., 2013; Logares et al., 2014) and can even occupy a disproportionally important role in biogeochemical cycles (Peter et al., 2011).

Given the strong variation in microbial community dynamics, it is highly likely that different factors control the dynamics of individual taxa, and thus, the way they are involved and embedded in the marine processes of microbial loop and food webs. Fungi, and their suite of different nutritional modes, are expected to be major players controlling taxon dynamics. Marine fungi are saprotrophs or symbionts, including pathogenic and mutualistic lifestyles (reviewed by Jones, 2000; Jobard et al., 2010). However, the biotic relationships of marine fungi go beyond direct interactions. For example, they can be members of cross-feeding networks, compete with other taxa for resources, and can produce allelopatic substances. It has also been shown that fungal tissue and spores constitute an important food source for a diversity of marine organisms (Newell et al., 1977; Cleary et al., 2016). In short, the role of fungi in the marine environment is extremely complex, and little understood.

Not only do we know little about the role of marine fungi, but we also do not understand their temporal dynamics. As visual identification of marine fungi is tedious and time consuming, there is a great need for molecular identification techniques to investigate the interplay of OTU dynamics and abundance (established as OTU-reads), and potential biotic interactions to increase our understanding on the impact these have on the planktonic community at large and even larger-scale ecosystem processes. We hypothesize that biotic interactions significantly influence the dynamics of individual OTUs and thus have effects on the entire mycoplankton community. Due to the diversity of possible biotic interactions we expect mycoplankton OTU dynamics to show very different patterns. We further assume that OTUs with similar abundance and temporal patterns have similar ecological niches, formed by biotic interactions. To test these hypotheses, we sampled seawater at the station Helgoland Roads in the German Bight (Wiltshire, 2004) over the course of 1 year on a weekly basis, correlated environmental data with Next Generation Sequencing-based fungal community data and calculated co-occurrence networks.

## Materials and Methods

### Sampling Scheme

Seawater samples were collected at the Long-Term Ecological Research (LTER) station “Kabeltonne” at Helgoland Roads (Wiltshire et al., 2010), located about 70 km off the mainland in the German Bight (Germany, 54° 11.3′ N, 7° 54.0′ E). Water samples were taken once a week (with the exception of days with adverse weather conditions) from July 2015 to June 2016, resulting in a total of 43 samples. Samples were collected on board of the research vessel *Aade*, with an impeller pump (Jabsco, United States) from ∼1 m-depth into a sterile 10 L bottle (Nalgene, Germany) and were directly processed in the laboratory of the Biologische Anstalt Helgoland. Two liters of the water was filtered directly without pre-filtration onto a Sterivex GP filter unit (0.22 μm PES membrane, Merck, Darmstadt, Germany) using a peristaltic pump (Verder, Germany) and stored at −20°C until further treatment.

The environmental data used to explain the dynamics patterns of the mycoplankton are part of the long-term monitoring program of the Biologische Anstalt Helgoland, collected at the same time point and water depth as samples for mycoplankton analysis (Wiltshire, 2004). This incorporated data on nutrients (SiO_4_, NO_2_, NO_3_, NH_4_, and PO_4_), salinity, dissolved organic matter (DOC), surface water temperature, water pH (Supplementary Table S1), seven phytoplankton groups, namely Dinophyceae (dinoflagellates), Coccolithophoridae INDeterminata (IND), flagellates, Bacillariophyceae (pennate diatoms), Bacillariales (pennate diatoms), Biddulphiales (centric diatoms), and Dictyochophyceae (silicoflagellates), as well as total phytoplankton cell counts (Wiltshire et al., 2015; Wiltshire, 2016; Sarker and Wiltshire, 2017) (Supplementary Table S2). Zooplankton sampling was carried out 3 days/week (for details, see Greve et al., 2004; Wiltshire et al., 2008) and counts of 42 different zooplankton groups were provided (Boersma et al., 2017).

### DNA Extraction, PCR and Sequencing

DNA was extracted using the Power Water DNA Isolation kit (MO BIO Laboratories, Dianova, Hamburg, Germany). After filtration, the Sterivex GP-filter unit was opened and the filter was transferred into an extraction vial. DNA was extracted following the manufacturer’s instructions. PCRs were performed using the fungi-specific primer pair nu-SSU-1334-5′/nu-SSU-1648-3′ (CGATAACGAACGAGACCT/ANCCATTCAATCGGTANT) (Vainio and Hantula, 2000), recently shown to be the most promising primer for marine fungal community analysis in environmental samples (Banos et al., 2018). Fungi-specific 18S rDNA gene sequence primers risk to co-amplify specific non-fungal eukaryotic organism groups. Therefore, the PCR protocol included the addition of four annealing blocking oligonucleotides. The blocking oligos with a 3′-amino linker C6 modification were specific to taxa within Stramenopiles (sequence of oligo: TCGCACCTACCGATTGAA), Alveolata (GTCGCTCCTACCGATTGA), Rhizaria (TTAA CGAACGAGACCTCGA) and *Telonema* (GACCTTAACC TACTAAATAGTTA) (Banos et al., 2018). PCR, library preparation and sequencing were performed at LGC Genomics GmbH, Berlin, Germany. All sequencing reactions were based upon an Illumina Miseq chemistry following the manufacturer’s instructions.

### Bioinformatics

Sequence reads were received in a demultiplexed form from which adapters and barcodes were already trimmed. Primers were removed using the software cutadapt v1.14 accepting an error tolerance of 16% (Martin, 2011). Forward and reverse reads were joined using PEAR v0.9.8 with the default settings (Zhang et al., 2013). The quality of sequence reads was checked by a 4-base wide sliding window approach embedded in the program Trimmomatic v 0.36 (Bolger et al., 2014). Sequences were excluded whenever the window average quality was lower than 20. Sequence reads shorter than 280 bp, or having more than 6 bp of homopolymer stretches, or more than four ambiguous symbols were deleted with Mothur v1.36.1 (Schloss et al., 2009). Finally, the cd-hit-dup tool of the software CD-HIT v4.7 (Li and Godzik, 2006; Fu et al., 2012) was used under the default settings to dereplicate the remaining sequence dataset and to detect chimeras with the *de novo* algorithm.

The sequence classification was based on a phylogenetic approach. In the first step, the dereplicated dataset was aligned to the non-redundant SILVA database SSURef_NR99_128 (Quast et al., 2013) using the SINA aligner v.1.2.11 (Pruesse et al., 2012) with the default settings. Based on the position of query sequences in the alignment, the SINA aligner classified the query sequences following the least common ancestor (LCA) rule. Only sequence reads classified as fungi under a 95% sequence threshold were extracted from the dataset for further analyses. Next, the extracted sequences were clustered into OTUs based on 98% sequence similarity using the CD-HIT-EST tool within the CD-HIT software. Singletons defined to be present only in a single sample with less than ten sequence reads were removed from the dataset. The final phylogenetic classification was done by phylogenetic placement of the sequences into the fungal phylogenetic reference tree (Yarza et al., 2017) using the Maximum Parsimony Algorithm, the standard algorithm of the ARB program v6.0.3 (Ludwig et al., 2004). Prior to the classification of the here generated sequences, the phylogenetic tree was further enriched by reference sequences of so far unrecognized soil-inhabiting order-level clades identified within the work of Tedersoo et al. (2017). The phylogenetic tree was then inspected for “novel fungal diversity” becoming obvious as newly formed clades. The clades were used as a working hypothesis to find different behavior patterns within the “novel diversity”. Thus, clades were manually assigned by transferring the taxonomic name of the tree branch but adding the word “clade” and increasing numeration in the case that more than one clade was found (e.g., Chytridiomycota Clade 01).

### Statistics

We were interested to understand if the patterns and responses to environmental factors of OTUs differed, depending on their abundance and temporal occurrence. Therefore, in addition to the dataset with information on all OTUs, we further created subsets of the community matrix based on OTU abundance and sample distribution (e.g., abundant, rare, resistent, transient OTUs): The dataset for abundant OTUs contained information of all OTUs, whose relative sequence abundance summed up to 90% of the one of the total community. The rare community fraction was formed by OTUs that were not included in the abundant community fraction. OTUs were defined as persistent OTUs, if they were present in at least 25 out of the 43 samples independent from their sequence abundance. In contrast, OTUs were defined as transient, if they occurred in ≤5 samples and represented at least 2% of the relative sequence abundance in the sample. Another sub-dataset contained information on all OTUs classified as zoosporic fungi. Zoosporic fungi exist as a motile spore using a flagellum for locomotion in at least one life stage, the so-called zoospore. Zoosporic fungi are discussed to play crucial roles in the dynamics of phytoplankton in oceans (Gutierrez et al., 2016; Lepere et al., 2016; Hassett et al., 2019). This is the reason why we investigated them as a group in more detail. Prior to any calculation, total OTU counts were Hellinger transformed (Bhattacharyya, 1943) and correlating environmental data were standardized using Z-score transformation (Clark-Carter, 2014). Generalized UniFrac distances (Chen et al., 2012) were calculated on community data and ordinated by principal component analysis (PCoA), the standard ordination with UniFrac-values (Lozupone and Knight, 2005). To test significance of the community response in a multivariate model, we first checked the candidate parameters for collinearity using a spearman correlation test. Parameters that had a correlation value higher than| for more than ten other factors were excluded from the analysis. Next, a distance-based redundancy analysis (dbRDA) was performed on generalized UniFrac distances. dbRDA was chosen as it can deal with the high variability found in microbial community datasets (for detailed information we refer to Legendre and Anderson, 1999). Important variables were detected using a forward selection method (function “ordistep” in vegan, see below). Analysis of variance was performed using the “anova.cca” command and permuting 999 times. Pearson correlation tests were used to test for possible interactions of the most abundant zoosporic fungal OTUs with the phytoplankton groups. The resulting p-values were adjusted for multiple testing with the false discovery rate (FDR) method (Benjamini and Hochberg, 1995).

All statistical analyses were carried out within the “R environment” v3.4.4 (R Core Team, 2015) using the packages “GUniFrac” v1.1 (Chen et al., 2012), “phyloseq” (McMurdie and Holmes, 2013), and “vegan” (Oksanen et al., 2013). The graphical representation of results was realized using the R package “ggplot2” (Wickham, 2016) and the program “GIMP2” version 2.8.22^1^.

### Co-occurrence Network Analyses

Co-occurrence network analyses were calculated with the weighted topological overlap (wTO) measure (Ravasz et al., 2002; Zhang and Horvath, 2005; Carlson et al., 2006). In contrast to a correlation-based approach, this measure allowed us to take all shared correlations between a pair of OTUs/parameters into account and to normalize it. The wTO measure was modified by Nowick et al. (2009) to accommodate both positive and negative interactions. Later, it was further modified to remove links incorporated by randomness by bootstrapping (Gysi et al., 2018). Thus, the wTO value is much more reliable for detecting links between a pair of OTUs/parameters and the nature of the interaction (±) than a correlation based network approach. As our data were based on a time series, the Blocked Bootstrap re-sample strategy was additionally applied. This strategy identifies blocks of high auto-correlations often occurring in the data distribution of time series by building an empirical distribution for each of the links (Efron and Tibshirani, 1993). This identifies blocks of highly auto-correlated time measures and builds an empirical distribution for each of the links. The wTO can be computed using this re-sampling method within the R package wTO (Gysi et al., 2018). All networks were constructed using the Pearson correlation, 1,000 bootstraps, the lag – autocorrelation window for the blocks – was defined using the autocorrelation function, and fixed in 4 weeks for all co-occurrence networks and in two for the inter-fungal interaction network. Networks were filtered for a Benjamini and Hochberg adjusted *P* < 0.001 and links were kept if their wTO weight were higher than |0.15|, minimum of the empirical quantile estimated within the “wTO” R package. We would like to point out that the wTO-values cannot be read as correlation values due to the different calculation methods applied. The temporal series underlying the dataset of this study, in which many of the fungal OTUs and/or environmental parameters do not exist in all time points, can cause as a side effect lower wTO-values. However, due to the robustness of the wTO approach, node interactions with lower wTO values are valid links that are included in the network calculation.

Observed differences in the interaction patterns (Fungus-Fungus, Fungus-Metadata, Fungus-Biota) and relationships (positive/negative) were tested for significance using the Chi-squared test and 1-sample proportion test, respectively.

## Results

### Mycoplanktonic Community Composition

After quality control, we retained 786,478 sequence reads classified as fungi. Three samples were removed from the dataset due to very low sequence output (time-points: 28.08.15, 17.09.15, 17.12.15). Fungal sequences clustered into 3,314 OTUs belonging to seven phyla (including undefined Basal fungi), 11 subphyla, 22 classes and 59 orders (Supplementary Tables S3, S4). Manual inspection of the phylogenetic tree allowed the identification of eight additional clades, three on the branch of the basal fungal lineages and three falling into the group of Cryptomycota (Rozellomycota) *sensu lato*, one Ascomycota, and one Basidiomycota clade (Supplementary Figure S1). None of the newly formed clades included a reference sequence of clades defined as novel diversity by Tedersoo et al. (2017). However, the closest neighbors to the Basal Fungi Clade 03 were Cryptomycota sequences of the *branch 2* and *GS11* clade.

**FIGURE 1:**
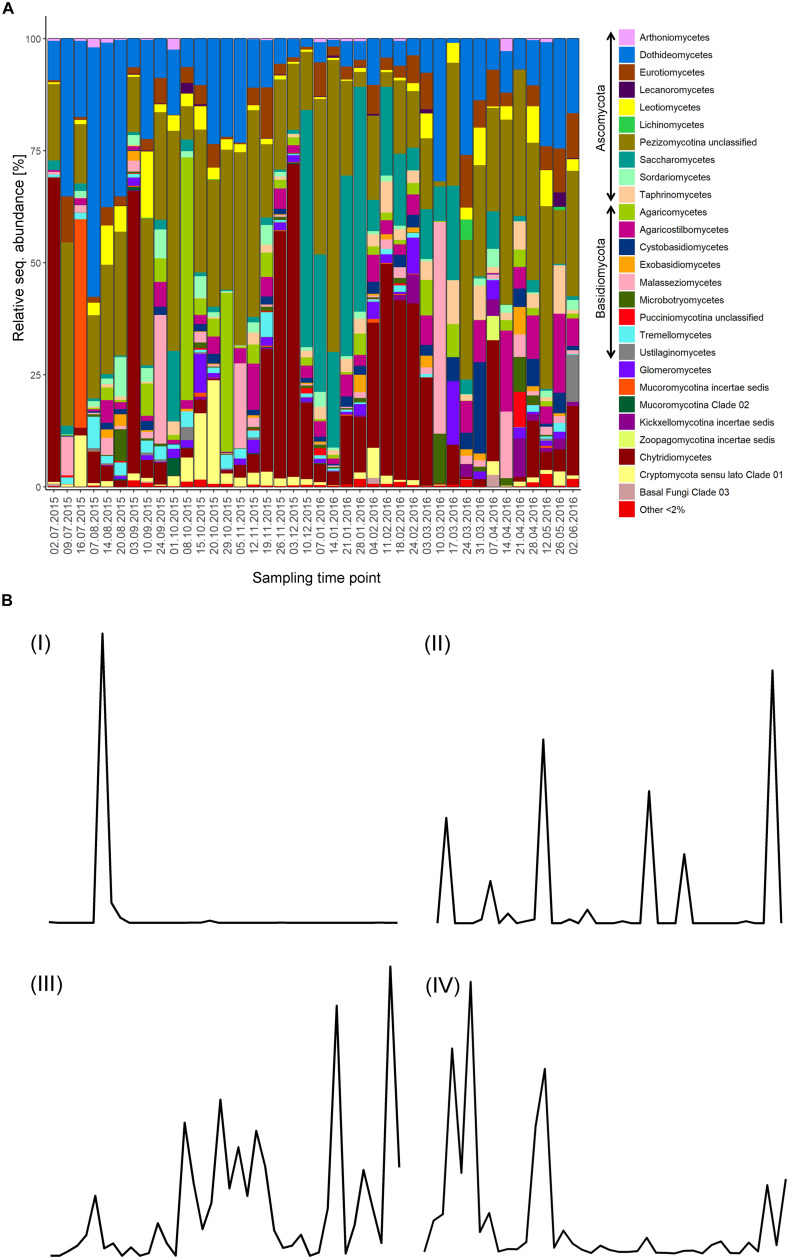
Mycoplankton community and OTU dynamics. **(A)** Taxonomic barcharts showing the relative sequence abundance of fungal classes for weekly taken surface water samples at the Helgoland Roads Station over the course of 1 year. **(B)** Examples for the four categories in which fungal OTUs were grouped based on their read abundance pattern. The following categories were defined: I, *Boom-bust like*; II, *Frequent peaking*; III, *Steady*; IV, *Long-lasting*. Fungal OTU dynamics were recorded over the course of 1 year starting in July 2015.

Ascomycota was the dominant phylum representing 24–88.5% of the relative sequence abundance for 31 out of the 40 samples. The majority of the Ascomycota sequences were phylogenetically assigned to unclassified Pezizomycotina, whilst a few samples were more represented by Saccharomycetes and Dothideomycetes. Chytridiomycota (primarily Chytridiomycetes) dominated the fungal community in six samples with 39–70% of the relative sequence abundance, and were otherwise only represented by 0–17% of the relative sequence abundance with a few exceptions. Basidiomycota were the most abundant phyla only in two samples, with 59 and 63% of the relative sequence abundance formed mainly by Agaricomycetes and Malasseziomycetes sequences. The time-point 16.07.15 was dominated by Mucoromycota sequences (47%), which exhibited, in general lower relative sequence abundance values (<5%) with few exceptions. Zoopagomycota and Cryptomycota *sensu lato* sequences were represented by less than 5% but increased at specific time-points with their relative sequence abundance to up to 23% (Figure 1A).

The abundance-based classification of the OTUs identified 128 abundant OTUs (3.9% of all OTUs). Most of them were assigned to Ascomycota (58 OTUs) and Basidiomycota (41 OTUs). Chytridiomycota, Cryptomycota *sensu lato*, Mucoromycota, and Zoopagomycota were represented with 13, 7, 6, and 3 OTUs, respectively. While the majority of Ascomycota were filamentous groups, the majority of Basidiomycota were classified as (dimorphic) yeasts. Dimorphic fungi have morphologically diverse life cycles, often including a yeast and a filamentous stage (Esser, 2014). The rare community contained a total of 3,186 OTUs (96.1% of all OTUs).

Regarding the occurrence of OTUs over the course of the sampling period, 28 OTUs were defined as persistent taxa (0.84% of all OTUs), from which three were part of the rare community. 19 persistent OTUs were classified as Ascomycota, seven as Basidiomycota, one as Chytridiomycota, and one as Mucoromycota. Out of the Dikarya OTUs, eight belonged to (dimorphic) yeast taxa and the rare persistent taxa were all filamentous Ascomycota.

The group of transient OTUs was composed of eight Basidiomycota, eight Chytridiomycota, six Ascomycota, two Zoopagomycota, and one OTU of the Basal Fungi Clade 03 forming a total of 25 OTUs (0.75% of all OTUs). Six of the transient OTUs were also categorized as highly abundant OTUs including three Chytridiomycota, one Zoopagomycota, one Basidiomycota, and one Ascomycota. Zoosporic fungi were represented with a total of 300 OTUs (9.1% of all OTUs) (Supplementary Tables S3, S5).

### Environmental Factors Structuring the Mycoplankton Community

The mycoplankton community structure was connected to diverse environmental factors (*n* = 34, *P* < 0.05, dbRDA, forward selection). Three abiotic factors were identified as significant in the analysis: temperature, NO_3_, and pH. Among the biotic factors, fish eggs, the larval stage of Echinodermata, the copepod *Centropages* spp., and the nauplius stage of copepods were described as factors connected with mycoplankton dynamics over a year (Table 1).

**TABLE 1:** Environmental factors with a significant effect on the fungal assemblage.

Environmental factor	*P*	*F*
*Zooplankton groups*		
Nauplius stage of copepods	0.049	1.51
*Centropages* spp.	0.025	1.65
Fish eggs	0.043	1.53
Larvae stage of Echinodermata	0.001	2.45
*Abiotic factors*		
Temperature	<0.001	4.05
NO_3_	0.01	1.84
pH	0.02	1.68

*To test significance of the multispecies response to environmental parameters, a distance-based redundancy analysis (dbRDA) was performed on generalized UniFrac distances (*n* = 34). Important variables were detected using the forward selection method. Analysis of variance was performed using the “anova.cca” command and permuting 999 times (vegan, R).*

Additionally, the PCoA shows a certain influence of seasonality on the phylogenetic structure of the mycoplankton community, especially for the samples taken in autumn (03/09/15-26/11/15) and winter (03/12/15-24/02/16) (Supplementary Figure S2).

### Patterns of Fungal OTU Dynamics

We investigated the patterns of the abundant fungal OTU dynamics in more detail, as they are the product of the interplay of the taxon’s life style and the relationship with environmental factors. Rare OTUs had to be excluded from this specific analysis, as their sequence abundance was often close to the detection limit. Based on the plotted read counts of individual fungal OTUs against time, four different categories of fungal OTU dynamics were identified and defined (Figure 1B): (I) The *Boom-bust like type* that is characterized by one to three peaks with a plateau phase lasting a maximum of 2 weeks. (II) The *Frequent peaking type*, which has numerous peaks (>3) and/or has a unique plateau phase of at least 3 weeks. (III) The *Steady type* which shows a continuous presence with fluctuations around a stable mean. (IV) The *Long-lasting type* is defined by at least two peaks with plateau phases of 2 weeks and longer. Many OTUs in this category showed distinct plateau phases of up to 6 weeks. 35 OTUs fell into the first category (I) while 38, 12, and 43 OTUs belonged to the category II, III, and IV, respectively. Only 50% of the persistent OTUs fell into the category III, which was solely formed by persistent OTUs. All transient OTU dynamics patterns were represented in category I. None of the abundant zoosporic OTUs were assigned to the category III (*Steady type*) (Supplementary Table S5).

**FIGURE 2:**
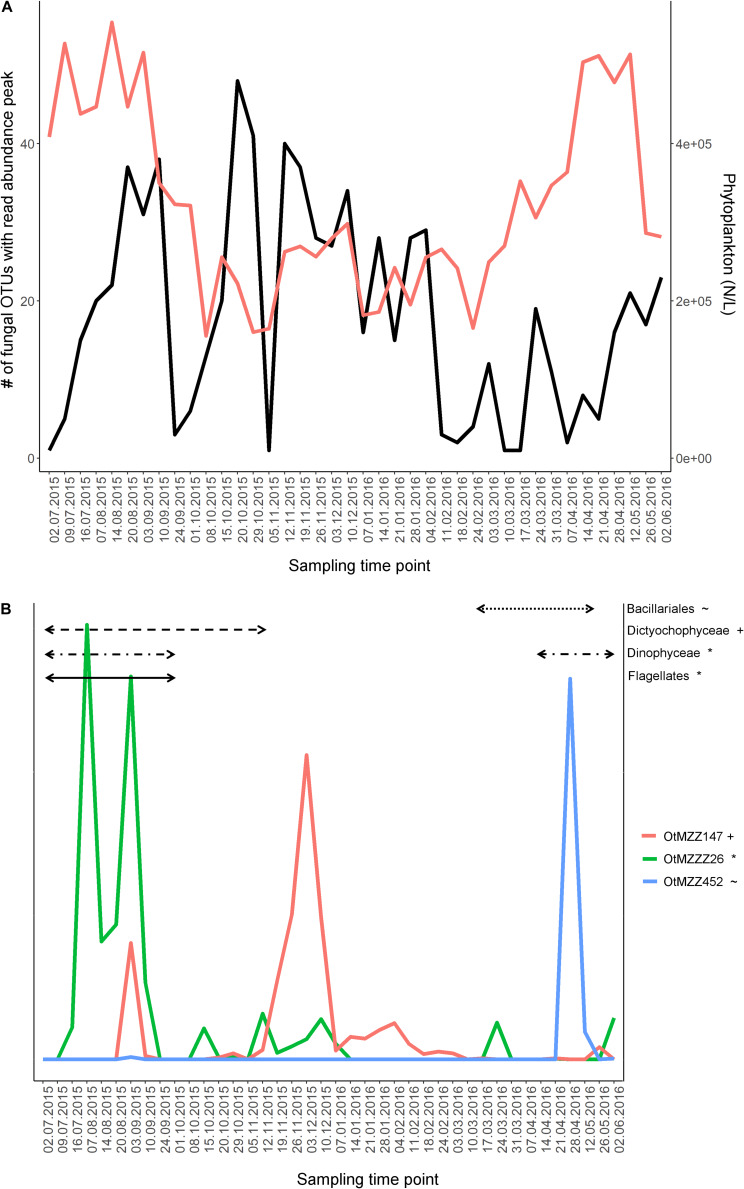
Fungal OTU dynamics in relation to phytoplankton dynamics. **(A)** Comparison of phytoplankton dynamics with fungal community dynamics. The variables used for comparison are total phytoplankton cell count and number of fungal OTUs peaking in read abundance at the respective time point. A peak in fungal OTU’s read abundance was defined for time points where the read abundance was higher than the averaged read abundance of the given OTU. Applied values for the fungi are the 128 abundant OTUs. Red line, count number of phytoplankton cells; black, number of fungal OTUs peaking at the given time-point. **(B)** Dynamics of three zoosporic fungal OTUs, potential parasites, over the course of 1 year. Time periods, in which their significantly correlated phytoplankton partner strongly increased in cell count numbers, are shown as horizontal black lines in the graph. Significance was tested using the Pearson correlation test (*n* = 40) adjusting the *p*-values with the false discovery rate method and filtering for *r* > |0.4|. The same symbol behind an OTU and a phytoplankton group indicate a significant relationship.

For the majority of sampling time points (70%) there were more than ten abundant fungal OTUs with a clear abundance peak, defined as an abundance value greater than the averaged value over all sampling time points for the specific OTU. Exceptions were samples taken on the 02.07.15, 09.07.15, 24.09.15, 01.10.15, 05.11.15, 11.-24.02.16, 10.-17.03.16, and 07.-21.04.16. Notably, dates with the highest numbers of fungal OTUs peaking (e.g., 20.-29.10.15 and 12.11.15-04.02.16) were the ones with the lower numbers of total phytoplankton cell counts (Figure 2A).

### The Fungus-Phytoplankton Relationships

Thirty out of the 128 abundant fungal OTUs (23%) were significantly correlated to one and up to several phytoplankton groups (pearson correlation, *n* = 40, FDR-adjusted *P* < 0.05, *r* > | 0.4|) including diverse Basidiomycota, Ascomycota, zoosporic fungi, and one Zoopagomycota OTU. From the seven tested phytoplankton groups, Bacillariales and Dinophyceae exhibited the highest number of correlations with fungal OTUs (with 7 and 6%, respectively) (Supplementary Table S5).

As for a large majority of zoosporic fungi, a parasitic life style has been described (Esser, 2014), we investigated the relationship among zoosporic OTUs’ dynamics and significantly correlated phytoplankton groups (*n* = 40, *P* < 0.05, *r* > |0.4|) in more detail, using the most abundant OTUs. Examples are the three Chytridiomycota *incertae sedis* OTUs OtMZZ147, OtMZZ452 and OtMZZ26. The OTU OtMZZ147 was significantly negatively correlated to Dictyochophyceae (*P* = 0.006; *r* = −0.43; *r*^2^ = 0.18) showing a first smaller peak during the Dictychophyceae bloom and a second time-retarded much larger peak. The peak observed for OTU OtMZZ452 matched the final stage of the significantly correlated Bacillariales bloom (*P* < 0.001; *r* = 0.85; *r*^2^ = 0.72). The dynamic of the OtMZZZ26 was correlated with both Dinophyceae (*P* < 0.001, *r* = 0.42; *r*^2^ = 0.18) and Flagellates (*P* < 0.001; *r* = 0.61; *r*^2^ = 0.38). The double peak observed for the fungal OTU fell into the bloom phase of both phytoplankton partners (Figure 2B).

### The Fungus-Zooplankton Relationships

**FIGURE 3:**
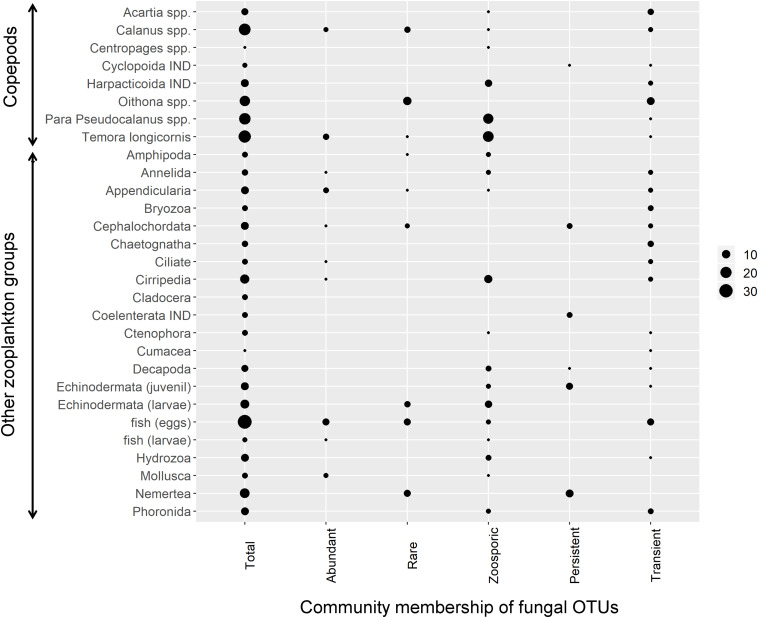
Negative zooplankton-fungus relationships. For each tested zooplankton group all significant negative links to fungal OTUs were counted across all calculated networks (Benjamini and Hochberg adjusted *P* ≤ 0.001; wTO > |0.15|). Circle size corresponds to the number of significant node links. The first column indicates the number of node links found for the total mycoplanktonc community while the other columns indicate node links to different fractions of the community, namely abundant, rare, zoosporic, persistent, and transient OTUs. IND, indeterminata.

**FIGURE 4:**
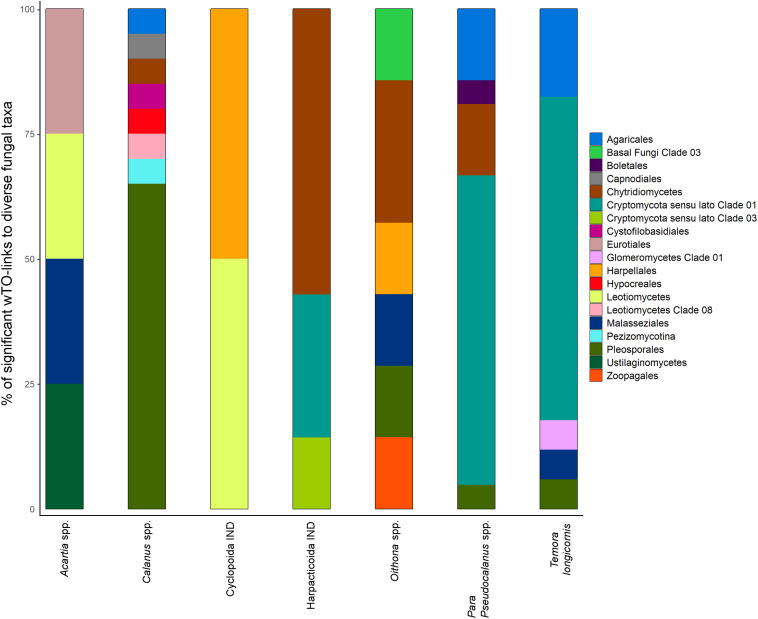
Stacked barcharts showing the taxonomic diversity of significantly related fungal partners with seven different copepod groups. Percentages are based on the counts of significant negative wTO links obtained from network analyses observed between fungal OTUs and copepods (Benjamini and Hochberg adjusted *P* ≤ 0.001; wTO > |0.15|).

Thirty out of the 42 zooplankton groups were significantly related with fungal OTUs (network analyses, Benjamini and Hochberg adjusted *P* ≤ 0.001; wTO > |0.15|). The number of observed relationships differed highly among zooplankton groups but always included zoosporic fungi and transient OTUs with few exceptions (Figure 3). The links between copepods and fungi were mainly negative (83%). The fungal assemblage differed greatly for different copepod genera/orders. Prominent fungal interaction partners were OTUs assigned to the zoosporic Chytridiomycetes *incertae sedis* and Cryptomycota *sensu lato* Clade 01, and the filamentous group of Pleosporales. Up to eight different fungal orders were negatively associated with a copepod group (Figure 4).

The majority of relationships to other zooplankton groups were negative (59%). The highest fungal diversity with 13 different fungal orders/undefined groups was observed with fish eggs, all being negative. Fungal interaction partners differed with zooplankton groups but no trend toward a phylogenetic group became visible (Supplementary Table S6).

### Signals of Potential Antagonistic Inter-Fungal Relationships

**FIGURE 5:**
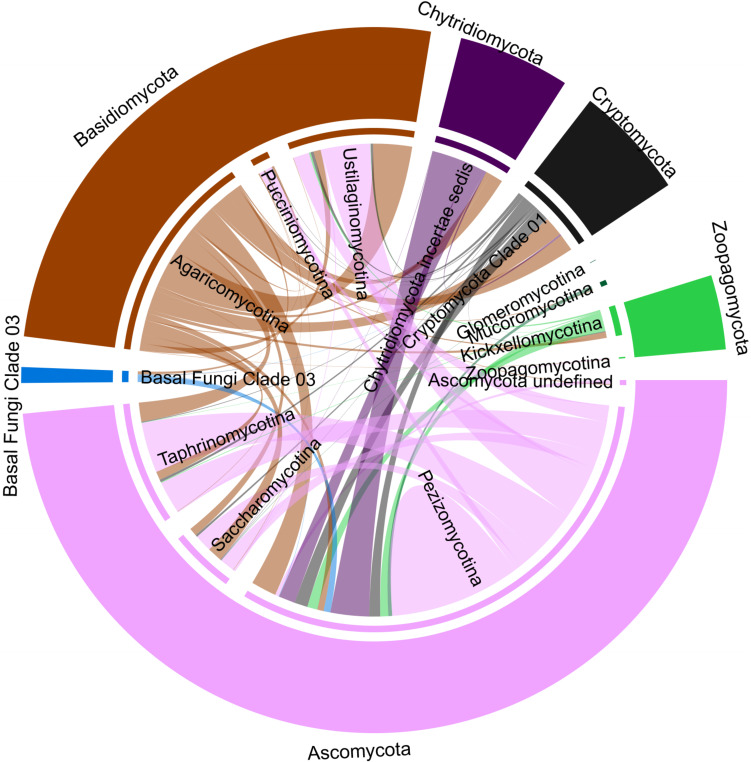
Chorddiagramm representing potential antagonistic interactions among fungi. The results are based on all significant negative correlations (Benjamini and Hochberg adjusted *P* ≤ 0.001; wTO > |0.15|) identified by network analysis using the dataset that comprised solely fungal OTUs. The thickness of the connecting lines signifies the number of correlated OTUs assigned to the two connected fungal taxa.

Thirty-seven percentage of the marine fungal OTUs showed potential antagonistic relationships with other fungi (network analysis, Benjamini and Hochberg adjusted *P* ≤ 0.001; wTO > |0.15|). Most of the significant negative associations were intra-phyla relationships dominated by Ascomycota and Basidiomycota. In contrast, all negative associations of zoosporic fungal OTUs showed exclusively an inter-phyla nature dominated by relationships to mainly Pezizomycotina and Agaricomycotina. Only 2% of all associations detected for the zoosporic fungi were between Cryptomycota and Chytridiomycota OTUs. The Basal fungi Clade 03 was represented by 1% of the negative associations related to Pezizomycotina and Agaricomycotina with only one exception. This kind of community membership had no impact on inter-fungal relationships (Figure 5).

### Co-occurrence Network

**FIGURE 6:**
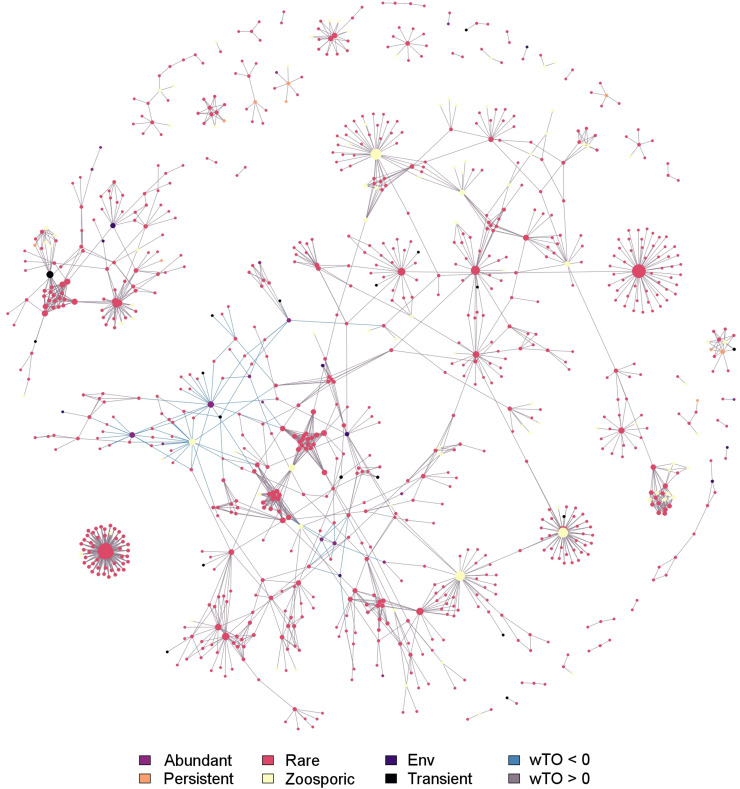
Co-occurrence network showing potential interactions among fungi and environmental parameters at Helgoland Roads. Network was filtered for a Benjamini and Hochberg adjusted *P* ≤ 0.001 and links were kept if its wTO weight were higher than |0.15|. The different community fractions, namely abundant, rare, persistent, transient and zoosporic OTUs, and environmental parameters (Env) are indicated by a color scheme. Lines connecting two nodes signify a significant link.

The network calculated for the whole fungal community, containing the environmental data, comprised 1,797 significant interactions formed by 937 fungal OTUs and 17 environmental factors (represented as nodes in the network, Benjamini and Hochberg adjusted *P* ≤ 0.001; wTO > |0.15|). With 95.2%, the large majority of interactions were significantly positive (1-sample proportion test, *P* < 0.05). The correlations were significantly dominated by fungus-fungus interactions (96.1%, Chi-Squared test, *P* < 0.05). In 53%, inter-phyla interactions were observed while the remaining 47% were intra-phyla interactions. Intra-phyla interactions were dominated by connections of Dikarya and, to a lesser extent, connections of other fungal phyla/clades. Among the nodes guaranteeing the network stability, members of Dikarya, Chytridiomycota and Cryptomycota *sensu lato* were represented. They belonged, with a few exceptions, to the rare community. Nine out of 28 persistent OTUs and ten out of 25 transient OTUs were part of the network but did not hold a prominent position within the network. The majority of nodes representing environmental factors were part of the main module except for fish eggs, Cirripedia, and the larvae of Echinodermata. The majority of interactions found between environmental data and fungal OTUs were negative (82.5%) (Figure 6 and Supplementary Table S6).

## Discussion

### Fungal Dynamics

This project aimed to identify potential marine fungal ecological relationships that shape fungal abundance patterns. The high diversity found among marine fungi requires investigations at a low taxonomic level, as this allows information on OTU abundance and temporary occurrence to be included in the analysis. Only if we understand the mechanisms that drive species dynamics, will we be able to decipher the mechanisms maintaining and modifying marine fungal community diversity. Since this study is based on Illumina tag sequencing data, it is difficult to estimate to what extent the signals are caused by dispersing spores, dormant structures, or by active members of the community. However, given (i) that the mycoplankton community was dominated by diverse phyla during the sampling period, (ii) that within the fungal OTU dynamics the increase/decrease of the sequence reads was sequential, (iii) that the patterns have been divided into only four categories due to similar temporal succession curves, and (iv) that these resemble already published patterns of aquatic microbes (Gerphagnon et al., 2013; Needham et al., 2013), we suppose that the majority of the sequence data is derived from taxa that actively respond to their environment.

About one-third of all fungal OTUs at Helgoland Roads were part of a dense co-occurrence network significantly dominated by positive relationships. Dense co-occurrence networks of microbial communities are normally advantageous for their members, as they generate an environment, in which specific biosynthetic pathways can be activated. This leads to an increase of the chemical diversity, which in return, stabilizes the community (Jousset et al., 2017; Oppong-Danquah et al., 2018). Rare OTUs mainly formed the observed co-occurrence patterns presumably acting as the backbone of the mycoplankton community. Such a backbone has large advantages, as the subcommunity formed represents a diversity reservoir that can respond quickly to environmental change on a temporal scale (Logares et al., 2014). Since ocean water is a highly dynamic system, strong fluctuations of environmental parameters occur over the course of the year. This could explain why roughly similar frequencies of inter- and intra-phyla relations were reported. Niche preferences are mainly visible at higher taxonomic levels (Philippot et al., 2010) and inter-phyla co-occurrence is favored under more constrained environmental conditions (Cao et al., 2018). Thus, both factors, inter-phyla relations and the high number of rare OTUs involved in forming the community backbone, can probably explain the mycoplankton diversity observed in this project.

The ecology of rare microbial subcommunities can sometimes differ substantially from that of more abundant microbes (Lynch and Neufeld, 2015), which may explain, why the abundant and persistent OTUs played a minor role for the overall network cohesion. This was also reflected in the dynamics patterns: 50% of all persistent OTUs fell into category III (*Steady type*), which in turn was formed only from persistent OTUs exclusively assigned to filamentous Ascomycota and (dimorphic) yeasts of the Dikarya. Marine yeasts are ubiquitous and the upper ocean layer can be inhabited by up to thousands of cells per liter of water (Meyers et al., 1967; Gerdts et al., 2004; Kutty and Philp, 2008), in which they can assimilate a large proportion of dissolved organic material (Suehiro et al., 1962). Both groups, (dimorphic) yeasts and filamentous Ascomycota, can be attached to larger particles (Jones, 2000; Bochdansky et al., 2017). Furthermore, it has recently been demonstrated that *Malassezia* (yeast) and *Cladosporium* (filamentous Ascomycota) taxa can use laminarin as food source (Cunliffe et al., 2017). Most marine Dikarya possess a wide spectrum of exo-enzymes (Raghukumar, 2008), so they are expected to be able to degrade various organic material of different decomposition stages and ages. These characteristics may explain the pattern of the type category III (*Steady type*) suggesting a saprophytic life style.

In contrast, all other OTUs, regardless of their taxonomy and morphology, showed dynamics pattern characterized by at least one very strong increase in read abundance. Only three of the nine abiotic factors, namely temperature, pH, and nitrate, were significantly associated with the dynamics of the fungal community. These factors are among those shaping mycoplankton assemblages independent of the sampling site in marine systems (Taylor and Cunliffe, 2016; Tisthammer et al., 2016; Duan et al., 2018). Zooplankton and phytoplankton groups, in contrast, were linked to fungal OTUs in numerous significant interactions.

Correlation tests and co-occurrence networks are important tools for the development of hypotheses on how marine fungi are embedded into marine food webs and microbial loops. However, highly complex data or data from time series may result in diverse errors in the detection of true relationships. We addressed these two points by using a wTO-based network analysis rather than a correlation-based one and by calculating time windows of possible data autocorrelation prior to the network analyses. Despite these tailored analyses, a side effect may be relative low wTO values, which is further enhanced if there are no replicates per sampling time-point. To identify the correct threshold for filtering the wTO values, the empirical distribution was used, computed by blocked bootstrapping and using the quantiles for the real data. A general drawback of correlation and network analyses is that it is unclear whether the detected relationship actually exists in reality and what its exact nature is. Correlations/wTO links can also simply occur because two species favor similar environmental conditions without any interaction. Nevertheless, strong real patterns of interactions are usually detected by these approaches (Krabberod et al., 2017).

Biotic interactions can be direct or indirect, and positive or negative. Positive associations may indicate that the interaction partners are organized in functional guilds or that interspecies cross-feeding exists. Negative associations are interpreted as antagonistic interactions, such as grazing, competition, predatory/pathogenic relations, or allelopathy. Biotic interactions can have marked effects on marine processes such as decomposition, nutrient cycling or energy flow between different trophic levels (Newell et al., 1977; Meunier et al., 2016). For this reason, we subsequently discuss the signals in our dataset for potential fungal interactions with phytoplankton, zooplankton, and between marine fungi in more detail.

### The Fungus-Phytoplankton Relationships

Phytoplankton blooms have a strong impact on the ecosystem because of succession and the fact that general growing conditions change dramatically over short periods of time. Due to the introduction of fresh organic material, one hypothesis would be that fungi, as heterotrophic organisms, benefit greatly from this and were seen to react as many OTUs with increased abundance. However, our data showed an opposite trend, so that the number of fungal OTUs with a strong read abundance peak was lowest during the spring phytoplankton bloom at Helgoland Roads. A similar trend was described by Duan et al. (2018) for a coastal ocean site in North Carolina, United States. It is difficult to draw conclusions from our data. However, this may indicate strong competitive pressure, which should be further investigated by a tighter sampling regime over the phytoplankton bloom and the addition of further correlative data such as bacterial cell count numbers. It also could be indicative of the fact that fresh marine plants and microalgae produce anti-bacterial and fungal substances, and that it is only when the environmental conditions decline for the phytoplankton parasites and degradative organisms can take hold (Naviner et al., 1999; Ianora and Miralto, 2010; Nuzzo et al., 2014).

Besides this general trend, several especially zoosporic OTUs showed significant relations to a phytoplankton group and their read abundance peaks were paralleled or slightly time-lagged to the one of the phytoplankton partner. The fungal dynamics patterns of these OTUs all fell into category I (*Boom-bust like type)* or II (*Frequent peaking type*) with the main peak lasting up to 4 weeks. These patterns correspond to the life cycles described for parasitic zoosporic freshwater fungi, which have similar time spans and abundance curves from the initial attachment of zoospores to host cells up to the final release of newly generated zoospores (Gerphagnon et al., 2013). The number of possible significantly associated hosts was different among the zoosporic fungi in our dataset. Host specificity is a matter of debate and both, broader and very narrow host specificity is discussed (Canter and Jaworski, 1982; De Bruin et al., 2004). Interestingly, the times of highest sequence abundance of the possible parasitic fungal OTUs were often not simultaneous with the times of highest cell numbers of the significantly correlating phytoplankton partner. However, this seems to be a frequently occurring phenomenon under natural conditions, since zoosporic parasitic fungal infection can be promoted at relative low host densities (Alster and Zohary, 2007).

### The Fungus-Zooplankton Relationships

The possible interactions with zooplankton were also dominated by negative associations. With respect to the marine food web structure and nutrient/carbon cycling this could be interpreted as top down control, where one organism increases in abundance to the detriment of the other. Under this assumption both, (i) fungi and (ii) zooplankton, can be the controlling force.

(i) Pathogenic or predacious fungi thrive on or prey on diverse zooplanktonic groups (Duddington, 1955; Barron, 1990; Nordbring-Hertz et al., 2006) and life stages (Ho and Perkins, 1985; Rasconi et al., 2011). Those fungi are phylogenetically highly diverse and can be found in all major fungal phyla (Duddington, 1956; Barron and Thorn, 1987; Ahren et al., 1998). Our results indicate a wide phylogenetic spectrum of possible pathogenic/predacious fungi in the mycoplankton of Helgoland Roads. Examples are Zoopagales OTUs, obligate parasites of smaller animals (Benny et al., 2014), or Harpellales OTUs, which are obligate gut symbionts with a parasitic life stage of crustaceans and arthropods (Lichtwardt, 2001; Wurzbacher et al., 2011). These fungal OTUs were classified as transient taxa, which was the fungal group being significantly associated to most of the tested zooplankton. Additionally, most of the fungal transient OTUs were related to more than one zooplankton group, which suggests that the potential interaction with the host/prey is not necessarily host specific. Among the fungal partners were also several of the rare OTUs, which may speak for an ecological importance of rare taxa, for example within food web structures or biotic interactions.

(ii) Fungi are enriched in polyunsaturated fatty acids and sterols (Kagami et al., 2007), which, for example, promote and upgrade the growth and reproduction of copepods (Breteler et al., 1999). In our dataset, the fungal interaction partners differed greatly among the copepod genera. This result may be interpreted as selective feeding of some copepod species (Pinto et al., 2001; Vargas et al., 2006), choosing prey that offer the right balance of nutrients or biochemical compounds (Meunier et al., 2016). Only one of the fungal persistent OTUs (mainly filamentous taxa), which was significantly related to zooplankton, was related to a copepod while the others were related to larger filter-feeding zooplankton. Food resource partitioning of zooplankton strongly depends on the feeding mechanism. While grazers, such as several copepods, strongly select for size and taxa (DeMott, 1986; Paffenhöfer, 1988), food uptake of other zooplankton is generally controlled over the particle size (Stuart and Klumpp, 1984). Furthermore, several copepods seem to discriminate between valuable (often living cells) and non-valuable particles (non-living) by rejecting or ingesting the latter ones at very low rates (Paffenhöfer and Van Sant, 1985). In contrast to free-floating single cells like zoospores or saprophytic yeasts, saprophytic marine filamentous fungal taxa seem to be attached to particles (Bochdansky et al., 2017). Following our hypothesis that fungi act as food source for zooplankton, it can be assumed that fungal persistent OTUs do not gain the same attention as food source for copepods as non-persistent fungi.

The high proportion of significantly negative associations found between zoosporic fungi and grazing zooplankton taxa underpins the possible existence of a marine mycoloop analogous to that in freshwater. Here, parasitic, zoosporic fungi infect large, inedible phytoplankton species. Assimilated phytoplankton-derived nutrients and organic material are transferred to grazing zooplankton via newly formed and released zoospores (Kagami et al., 2014). It can be assumed that such a fungal based trophic bridging of phytoplankton and zooplankton affects the marine carbon flux and subsequent functioning of the carbon cycle (Amend et al., 2019).

### Potential Antagonistic Inter-Fungal Relationships

Thirty-seven percent of the fungal OTUs were significantly negatively related with another fungal OTU, which may indicate negative inter-fungal interactions as described for many fungal taxa (Laughlin and Spatafora, 2014). In the context of antagonistic relations, a negative association may indicate mycoparasitism, competition, resource partitioning, or allelopathy. The patterns of potential negative associations differed among Dikarya and Basal Fungi in our study: Thus, the dimorphic and yeast groups of Dikarya were mainly involved in negative intra-phyla associations, a pattern that Ruszkiewicz-Michalska (2010) has described for mycoparasitic Dikarya. For dimorphic taxa, the filamentous stage is often the one that infects and explores the host’s resources while the yeast stage feeds saprotrophically (Sampaio, 2004). The antagonistic nature of several yeasts within the Saccharomycetales and Tremellales is different: They secret fungicidal protease-sensitive toxins (Golubev, 1998). The effect on the counterpart is highly specific and depends for example on its sexual, chemotaxonomic and physiological characteristics (Golubev and Nakase, 1997; Golubev, 2013).

In contrast to the Dikarya, the negative interactions of the Basal Fungi were nearly exclusively of an inter-phyla nature. Thus, the related partners of several OTUs of the Cryptomycota *sensu lato* Clade 01 were mainly Dikarya or Chytridiomycota. Cryptomycota are parasites of all kinds of organisms (Jones et al., 2011). For example, some *Rozella* species infect diverse Chytridiomycota and act as necrotrophs, degenerating their cytoplasm for nutrient uptake (Boddy, 2015). Other notable relationships involved the taxa assigned to the Basal Fungi Clade 03. This clade branched on the basal part of the fungal phylogenetic reference tree. Phylogenetic relations among Basal Fungi are still under debate and inclusion into the phylum of Microsporidia has been proposed (Bass et al., 2018). Microsporidia are obligate and highly efficient parasites and include mycoparasites. They sporulate abundantly when cultured with their hosts (Ahrendt et al., 2018). In our dataset, most of the possible antagonists exhibited category I (*Boom-bust like type*) or III (*Steady type*) dynamic patterns. Our hypothesis for the occurrence of these opposite patterns is that antagonistic fungi have different ranges of host specificity or that the interaction is strongly influenced by external environmental parameters.

## Conclusion

Our data suggest that biotic interactions are important for the niche formation of fungi in marine surface water. The kind of interaction seems to be related to fungal OTU abundance and its temporal occurrence. Our results further suggest a strong antagonistic relationship between fungi and other planktonic organisms. In an ecological context this may mean (i) a top-down control by fungi on phytoplankton or zooplankton populations with consequences for the microbial loop and food web structure; (ii) that fungi transfer organic material and nutrients to other planktonic organisms by acting as food source (one possible scenario may be a marine mycoloop); and (iii) that the proliferation of individual fungal species harmful to other planktonic organisms may be limited by other fungal species.

To further build upon this work, one to one interactions between individual fungal OTUs and plankton organisms should be analyzed by modern fluorescent microscopy to decipher the true nature of interactions and OTU dynamics. This approach should be accompanied with a genetic analysis *in vitro* culturing in order to unravel the mechanisms which are essential to the interaction.

## Data Availability Statement

The datasets generated for this study can be found in the Sequence data can be obtained from the European Nucleotide Archive (ENA^2^) with the accession number PRJEB33370. The following corresponding metadata is published in PANGAEA^3^: The count data of mesozooplanktonic individuals can be accessed over (Boersma and Renz, 2016; DOI: 10.1594/PANGAEA.864586) and (Boersma and Renz, 2017; DOI: 10.1594/PANGAEA.870606) for the years 2015 and 2016, respectively. The cell count data of the nine phytoplankton groups for the year 2015 can be accessed over (Wiltshire, 2016; DOI: 10.1594/PANGAEA.862909). Exceptions are the data of the hydrochemistry and DOC as well as the phytoplanktonic cell counts for the year 2016, which have not been submitted yet and can thus be accessed over the Supplementary Tables S1, S2, respectively. The fully annotated OTU table can be found as Supplementary Table S3 and the related representative sequences in Supplementary Table S4.

## Author Contributions

MR, GG, AW, and FG planned and designed the study. KW provided and interpreted physiochemical and phytoplankton biodiversity data. MB provided and interpreted DOC and zooplankton biodiversity data. SB, MR, and TR-H analyzed the fungal biodiversity data. DG run and interpreted the co-occurrence networks. SB and MR wrote the manuscript. All authors have reviewed and approved the manuscript.

## Conflict of Interest

The authors declare that the research was conducted in the absence of any commercial or financial relationships that could be construed as a potential conflict of interest.

## Footnotes

1 https://www.gimp.org

## Supplementary Material

FIGURE S1 Positioning of the newly identified clades within the fungal phylogenetic tree used for the classification of fungal sequences. The newly formed clades are only composed by sequences generated in this study (clades are colored in red). Clades were collapsed at the branch, which defines them. The phylogenetic tree is based on the fungal reference tree of Yarza et al. (2017).

FIGURE S2 Principal coordinate analysis (PCoA)on mycoplankton community data. A generalized UniFrac distance matrix was generated revealing phylogenetic differences of fungal assemblages over sampling time-points. The color code defines the four seasonsas described in Lucas et al. (2015). In short, spring (1 March–31 May); summer (1 June-31 August); autumn (1 September–30 November); winter (1 December–29 February).

FIGURE S3 Dynamics of phytoplankton abundance (cell count) over the course of a year (summer 2015 to summer 2016).

TABLE S1 Hydrochemistry and DOC content at Helgoland Roads for the different sampling time points.

TABLE S2 Phytoplankton cell counts at Helgoland Roads for the different sampling time-points.

TABLE S3 Fully annotated fungal OTU table with read abundance values over the different sampling time-points. Reference sequences for individual OTUs can be found in Supplementary Table S4.

TABLE S4 Fasta-file with representative sequences of all annotated OTUs found in Supplementary Table S3.

TABLE S5 Characteristics of fungal OTUs (without rare OTUs). Information includes community membership, relative sequence abundance, phylogenetic placement, numbers of samples being present, pattern type (dynamic), significant correlation withphytoplankton groups and time-points of peaking in sequence abundance (Pearson correlation: *n* = 40, FDR-adjusted *P* < 05; *r* > |0.4|; *r* ^2^).NA, no taxonomic information available.

TABLE S6 Significant node relations as identified by network analyses.Benjamini& Hochberg adjusted *P* ≤ 0.001; wTO > |0.15|; note, the wTO is not a correlation value (we refer to the MM section). put on bold text must link.
