# Supplementary material for: Seasonal Dynamics of Pelagic Mycoplanktonic Communities: Interplay of Taxon Abundance, Temporal Occurrence, and Biotic Interactions
Source: Front Microbiol. 2020 Jun 26;11:1305. doi: 10.3389/fmicb.2020.01305 (PMC7333250; doi:10.3389/fmicb.2020.01305)

Supplementary Figure 1: Positioning of the newly identified clades within the fungal phylogenetic tree used for the classification of fungal sequences. The newly formed clades are only composed by sequences generated in this study (clades are colored in red). Clades were collapsed at the branch, which defines them. The phylogenetic tree is based on the fungal reference tree of Yarza et al. (2017).

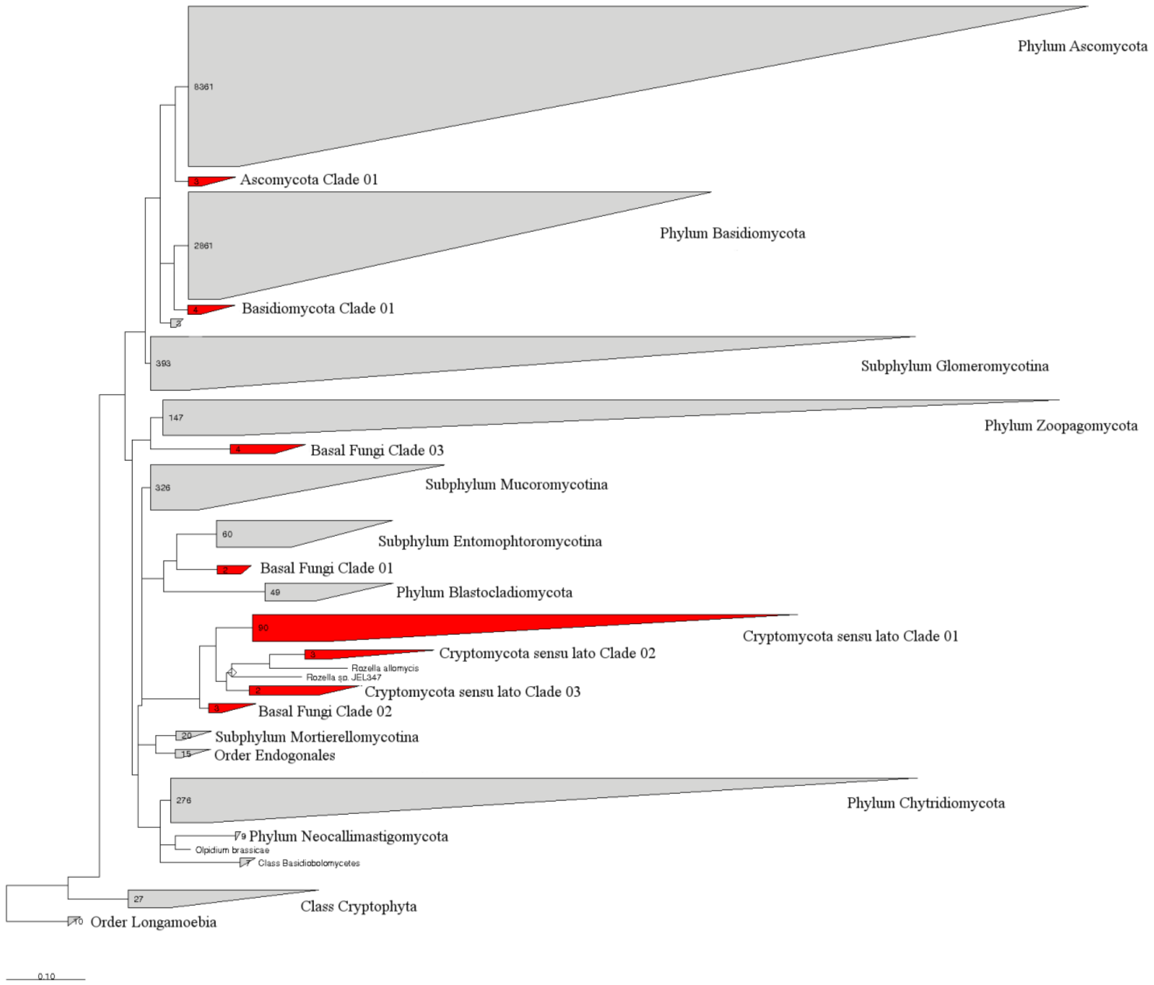

Supplement: FIGURE S1 — Positioning of the newly identified clades within the fungal phylogenetic tree used for the classification of fungal sequences. The newly formed clades are only composed by sequences generated in this study (clades are colored in red). Clades were collapsed at the branch, which defines them. The phylogenetic tree is based on the fungal reference tree of Yarza et al. (2017). [file Data_Sheet_1.PDF]
