# Supplementary material for: Seasonal Dynamics of Pelagic Mycoplanktonic Communities: Interplay of Taxon Abundance, Temporal Occurrence, and Biotic Interactions
Source: Front Microbiol. 2020 Jun 26;11:1305. doi: 10.3389/fmicb.2020.01305 (PMC7333250; doi:10.3389/fmicb.2020.01305)

Supplementary Figure 2: Principal coordinate analysis (PCoA) on mycoplankton community data. A generalized UniFrac distance matrix was generated revealing phylogenetic differences of fungal assemblages over sampling time-points. The color code defines the four seasons as described in Lucas et al. (2015). In short, spring (1 March–31 May); summer (1 June–31 August); autumn (1 September–30 November); winter (1 December–29 February).

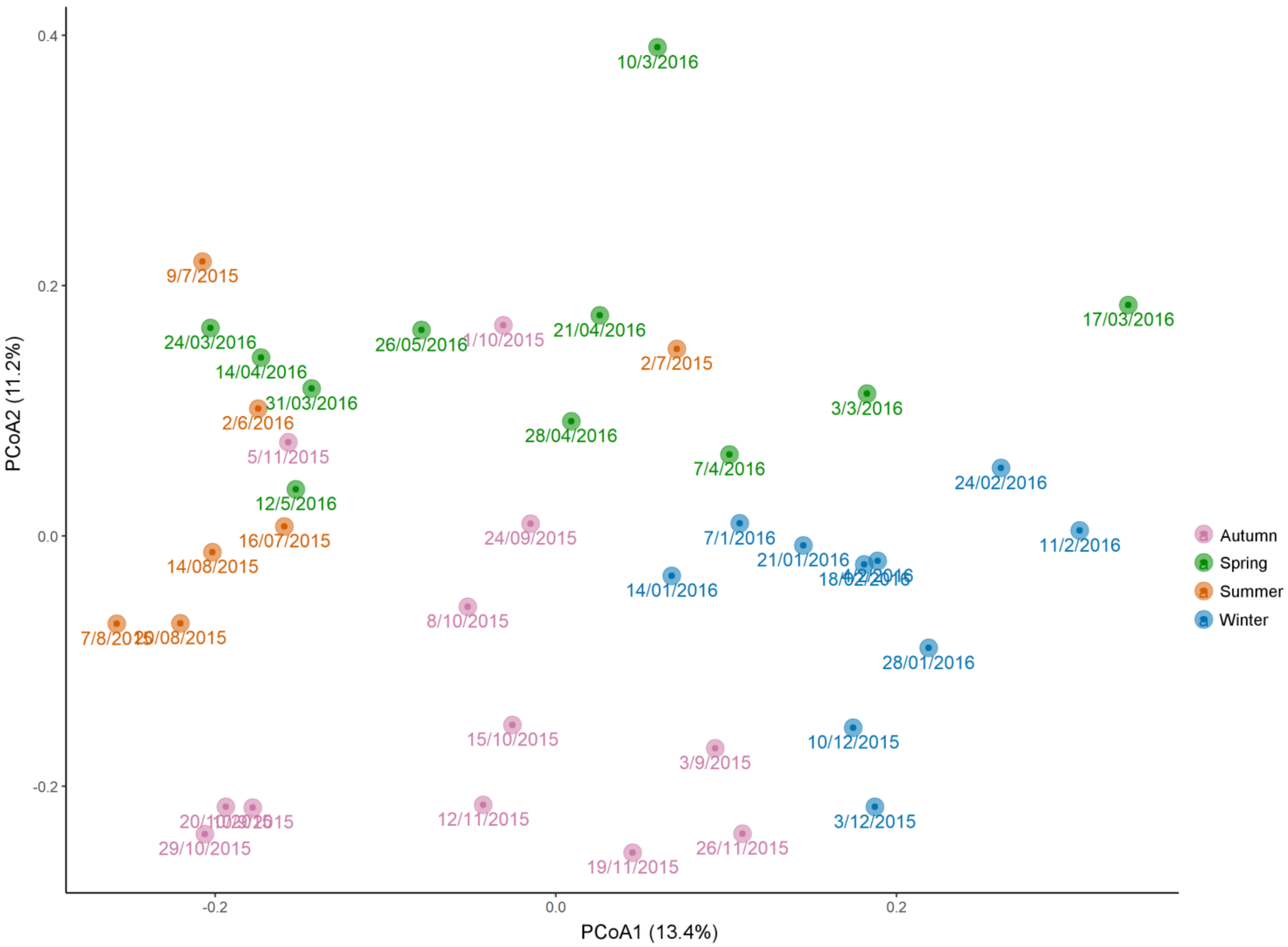

Supplement: FIGURE S2 — Principal coordinate analysis (PCoA)on mycoplankton community data. A generalized UniFrac distance matrix was generated revealing phylogenetic differences of fungal assemblages over sampling time-points. The color code defines the four seasonsas described in Lucas et al. (2015). In short, spring (1 March–31 May); summer (1 June-31 August); autumn (1 September–30 November); winter (1 December–29 February). [file Data_Sheet_2.PDF]
