# Supplementary material for: Seasonal Dynamics of Pelagic Mycoplanktonic Communities: Interplay of Taxon Abundance, Temporal Occurrence, and Biotic Interactions
Source: Front Microbiol. 2020 Jun 26;11:1305. doi: 10.3389/fmicb.2020.01305 (PMC7333250; doi:10.3389/fmicb.2020.01305)

Supplementary Figure 3: Dynamics of phytoplankton abundance over the course of a year (summer 2015 to summer 2016).

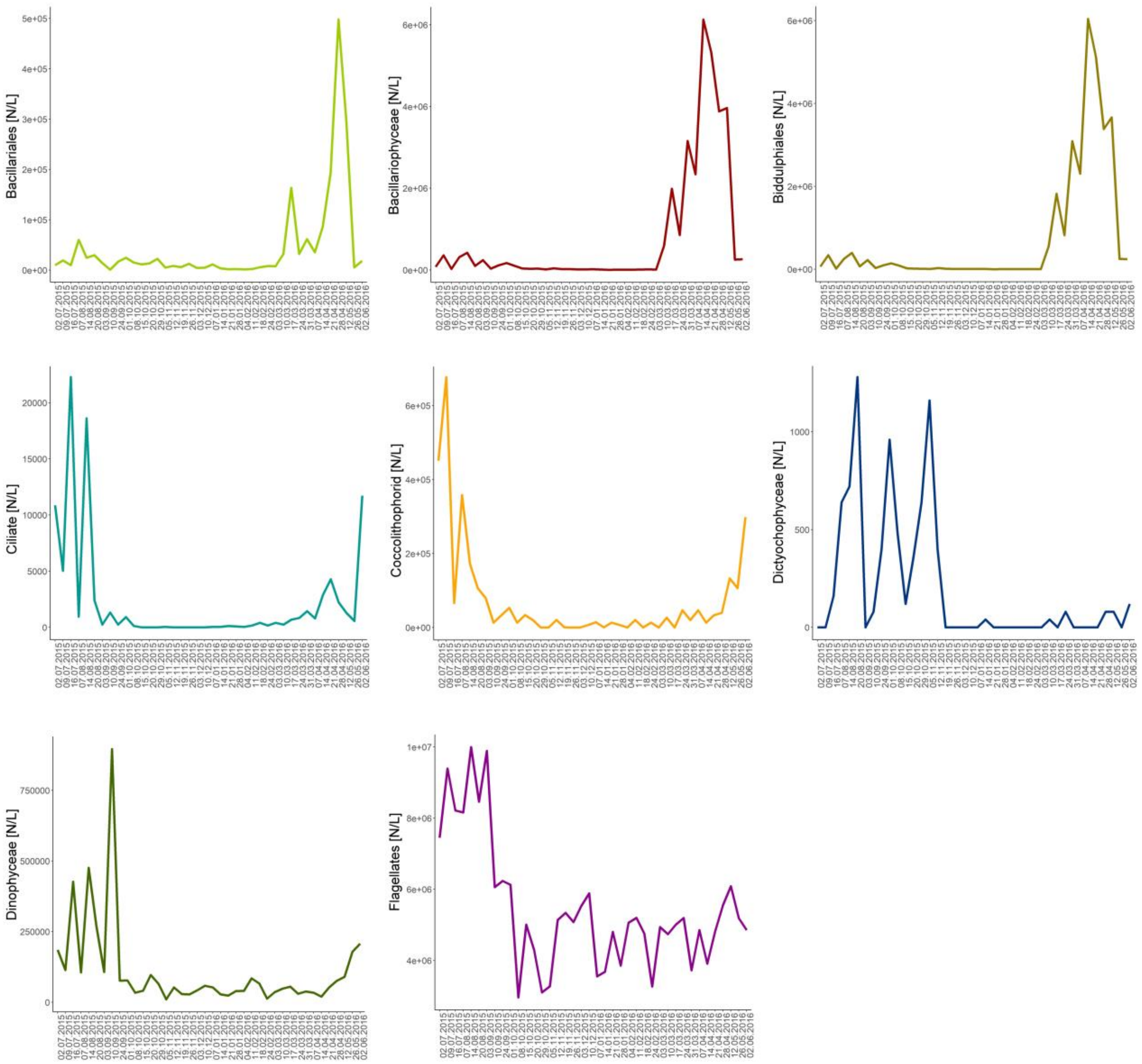

Supplement: FIGURE S3 — Dynamics of phytoplankton abundance (cell count) over the course of a year (summer 2015 to summer 2016). [file Data_Sheet_3.PDF]
